# Triage systems in low-resource emergency care settings

**DOI:** 10.2471/BLT.23.290863

**Published:** 2025-01-30

**Authors:** Rob Mitchell, Gerard O’Reilly, Colin Banks, Garry Nou, John Junior McKup, Carl Kingston, Mangu Kendino, Donna Piamnok, Peter Cameron

**Affiliations:** a School of Public Health and Preventive Medicine, Monash University, 553 St Kilda Road, Melbourne, Victoria 3004, Australia.; b Townsville University Hospital, Townsville, Australia.; c National Department of Health, Port Moresby, Papua New Guinea.; d Mount Hagen Provincial Hospital, Mount Hagen, Papua New Guinea.; e Port Moresby General Hospital, Port Moresby, Papua New Guinea.; f West Sepik Provincial Health Authority, Vanimo, Papua New Guinea.

## Abstract

Triage is widely regarded as a core emergency care function, as reflected in the World Health Organization (WHO) *Emergency care systems framework* and in recent World Health Assembly resolutions. In this article, we explore the evidence supporting triage in low-resource settings, with a focus on the *Interagency Integrated Triage Tool*. Following its release by WHO in the early stages of the coronavirus disease pandemic, the tool has been implemented across a range of low- and middle-income countries. We report evidence regarding its acceptability and performance from Papua New Guinea in the WHO Western Pacific Region. Data from four single-centre studies suggest that the tool can be reliably and efficiently applied by health workers, and its predictive validity is within the performance range of other triage instruments. The system is highly regarded by emergency care clinicians, and can be implemented with limited digital or in-person training. Although triage has intuitive and widely acknowledged value, recent research has identified a lack of high-quality evidence supporting an association between triage implementation and improved clinical outcomes. Evidence from several pre-post intervention studies suggests that the introduction of triage can reduce waiting times and mortality, but these data may have been subject to confounding and publication bias. Further research is required to establish the performance characteristics of the *Interagency Integrated Triage Tool* in other countries and contexts, and more rigorously examine the impact of triage implementation on quality of care.

## Résumé

Le triage est largement considéré comme une fonction essentielle des soins d’urgence, comme en témoignent le *cadre des systèmes de soins d’urgence* de l’Organisation mondiale de la santé (OMS) et les récentes résolutions de l’Assemblée mondiale de la santé. Le présent article examine les données probantes à l’appui du triage dans les environnements à faibles ressources, en mettant l’accent sur l’outil de triage intégré interagences (*Interagency Integrated Triage Tool*). Depuis sa publication par l’OMS au début de la pandémie de coronavirus, cet outil a été mis en œuvre dans plusieurs pays à revenu faible ou intermédiaire. Nous présentons des données probantes concernant son acceptabilité et ses performances en Papouasie-Nouvelle-Guinée, dans la région du Pacifique occidental de l’OMS. Les données de quatre études monocentriques suggèrent que cet outil peut être appliqué de manière fiable et efficace par les agents de santé et que sa validité prédictive se situe dans la plage de performance d’autres instruments de triage. Le système est fortement apprécié des cliniciens d’urgence et peut être mis en œuvre avec une formation en ligne ou présentielle limitée. Bien que la valeur intuitive du triage soit largement reconnue, des recherches récentes ont identifié un manque de données probantes de qualité soutenant une association entre la mise en œuvre du triage et l’amélioration des résultats cliniques. Les données de plusieurs études de pré- et post-hospitalisation suggèrent que l’introduction du triage est de nature à réduire les temps d’attente et la mortalité. Toutefois, ces données peuvent avoir été sujettes à des facteurs de confusion et à des biais de publication. Des recherches supplémentaires sont nécessaires pour établir les caractéristiques de performance de l’outil de triage intégré interagences dans d’autres pays et contextes ainsi que pour examiner plus rigoureusement l’impact de la mise en œuvre du triage sur la qualité des soins.

## Resumen

El triaje está ampliamente considerado como una función básica de la atención de urgencias, tal y como se refleja en el *marco de los sistemas de atención de urgencias* de la Organización Mundial de la Salud (OMS) y en recientes resoluciones de la Asamblea Mundial de la Salud. En este artículo, se exploran las pruebas que apoyan el triaje en entornos de bajos recursos, centrándose en la herramienta de triaje integrado entre agencias (*Interagency Integrated Triage Tool*). Tras su lanzamiento por la OMS en las primeras fases de la pandemia de coronavirus, la herramienta se ha aplicado en diversos países de ingresos bajos y medios. Se presentan pruebas de su aceptabilidad y rendimiento en Papúa Nueva Guinea, en la Región de la OMS para el Pacífico Occidental. Los datos de cuatro estudios realizados en un único centro indican que los profesionales sanitarios pueden aplicar la herramienta de forma fiable y eficiente, y que su validez predictiva está dentro del rango de rendimiento de otros instrumentos de triaje. Los médicos de urgencias valoran muy positivamente el sistema, que puede implementarse con una formación digital o presencial limitada. Aunque el triaje tiene un valor intuitivo y ampliamente reconocido, investigaciones recientes han identificado una falta de pruebas de alta calidad que respalden una asociación entre la implementación del triaje y la mejora de los resultados clínicos. Las pruebas de varios estudios de intervención previos y posteriores sugieren que la introducción del triaje puede reducir los tiempos de espera y la mortalidad, aunque es posible que estos datos estén sujetos a factores de confusión y a sesgos de publicación. Se requieren más investigaciones para establecer las características de rendimiento de la herramienta de triaje integrado entre agencias en otros países y contextos, y examinar de forma más rigurosa el impacto de la implementación del triaje en la calidad de la atención.

## ملخص

يُنظر إلى الحجر الصحي على نطاق واسع باعتباره وظيفة أساسية في رعاية الطوارئ، كما يظهر في إطار عمل أنظمة رعاية الطوارئ التابع لمنظمة الصحة العالمية (WHO)، وفي قرارات جمعية الصحة العالمية الأخيرة. في هذه المقالة، نستكشف الأدلة التي تدعم الحجر الصحي في البيئات ذات الموارد المنخفضة، مع التركيز على أداة الحجر الصحي المتكاملة بين الوكالات. بعد إصدارها بواسطة منظمة الصحة العالمية في المراحل المبكرة من جائحة مرض فيروس كورونا، تم تنفيذ الأداة عبر مجموعة من الدول ذات الدخل المنخفض والدخل المتوسط. نحن نقدم أدلة بشأن مدى قبولها وأدائها من بابوا غينيا الجديدة، في منطقة غرب المحيط الهادئ التابعة لمنظمة الصحة العالمية. تشير البيانات من أربع دراسات أجريت في مركز واحد إلى أنه يمكن للعاملين في القطاع الصحي تطبيق الأداة بشكل موثوق وفعال، وأن صلاحيتها التنبؤية تقع ضمن نطاق أداء أدوات الحجر الصحي الأخرى. يحظى النظام بتقدير كبير من جانب أطباء رعاية الطوارئ، ويمكن تنفيذه بتدريب رقمي أو شخصي محدود. على الرغم من أن الحجر الصحي له قيمة بديهية ومعترف بها على نطاق واسع، فقد حددت الأبحاث الحديثة نقصًا في الأدلة عالية الجودة التي تدعم الارتباط بين تنفيذ الحجر الصحي وتحسين النتائج الإكلينيكية. تشير الأدلة المستمدة من العديد من الدراسات التي أجريت قبل وبعد التدخل إلى أن إدخال الحجر الصحي يمكن أن يقلل من أوقات الانتظار ومعدل الوفيات، ولكن هذه البيانات ربما تكون تعرضت للخلط والتحيز في النشر. هناك حاجة إلى مزيد من البحث لتحديد خصائص أداء أداة الحجر الصحي المتكاملة بين الوكالات في دول وأوضاع أخرى، وفحص تأثير تنفيذ الحجر الصحي على جودة الرعاية بشكل أكثر فعالية.

## 摘要

正如世界卫生组织 (WHO) 急诊护理环境系统框架和世界卫生大会最近达成的决议所示，分诊被广泛认为是一项重要的急诊护理功能。在本文中，我们重点围绕机构间综合分诊工具，探讨了支持在资源匮乏环境中实施分诊系统的证据。自世界卫生组织在冠状病毒病大流行疫情的早期阶段发布该工具以来，一系列中低收入国家已实施了该工具。我们报告了世界卫生组织西太平洋区域巴布亚新几内亚提供的其可接受性和性能相关证据。来自四项单一中心研究的数据表明，该工具具有可靠性和高效性，可供卫生工作者放心使用，且其预测效度在其他分诊工具的性能范围内。该系统可通过有限的数字或现场培训来实施，受到急诊急救临床医生的高度评价。尽管分诊具有直观和广为人知的价值，但最近的研究发现，缺乏可有效表明实施分诊系统与临床结果改善之间存在关联的高质量证据。来自多项对干预前后研究的证据表明，引入分诊系统可以减少等待时间并降低死亡率，但这些数据可能存在混杂和发表偏倚。需要进行进一步研究以确定机构间综合分诊工具在其他国家中和不同背景下的性能特征，并以更严格的标准评估实施分诊系统对护理质量的影响。

## Резюме

Сортировка пациентов широко рассматривается как одна из основных функций оказания неотложной помощи, что отражено в *Рамочном подходе к системам оказания неотложной помощи* Всемирной организации здравоохранения (ВОЗ) и в последних резолюциях Всемирной ассамблеи здравоохранения. В данной статье анализируются данные, подтверждающие необходимость проведения сортировки пациентов в условиях недостатка ресурсов, при этом особое внимание уделяется межведомственному объединенному способу сортировки. После того как этот способ был обнародован ВОЗ на ранних стадиях пандемии коронавирусной инфекции, его внедрили в ряде стран с низким и средним уровнем дохода. Авторы приводят данные о его приемлемости и эффективности в Папуа – Новой Гвинее, Западно-Тихоокеанском регионе ВОЗ. Результаты четырех одноцентровых исследований свидетельствуют о том, что этот способ может надежно и эффективно применяться медицинскими работниками, а его прогностическая валидность находится в диапазоне эффективности других способов сортировки пациентов. Система высоко ценится врачами скорой помощи и может быть внедрена путем ограниченного обучения с применением цифровых технологий или очного обучения. Несмотря на интуитивную и общепризнанную ценность сортировки пациентов, результаты последних исследований свидетельствуют об отсутствии высококачественных доказательств, подтверждающих связь между внедрением такой сортировки и улучшением клинических результатов. По данным нескольких исследований, проведенных до и после вмешательства, введение сортировки может сократить время ожидания и смертность, однако на эти данные могли повлиять некоторые вмешивающиеся факторы и предвзятость публикаций. Для определения характеристик эффективности межведомственного объединенного способа сортировки в других странах и условиях, а также для более тщательного изучения влияния внедрения сортировки на качество медицинской помощи необходимо проведение дальнейших исследований.

## Introduction

Triage aims to identify and prioritize patients with time-sensitive care needs, and fulfils a critical role whenever demands for care exceed the available resources.^1^^,^^2^ Regarded as a core emergency care function,^3^ triage is an important tool for ensuring the fair and efficient use of health-care resources.^1^^,^^2^ The global relevance of triage has been recognized in the World Health Organization (WHO) *Emergency care systems framework*^4^ and in several resolutions from the World Health Assembly.^5^^–^^7^

In health-care facilities, a typical approach to triage involves a clinician assigning a category of urgency to all patients who present with emergency care needs. This categorization is then used to determine the order of patient assessment.^1^^,^^8^ Importantly, triage is distinct from screening. In the emergency care context, this refers to the assessment of communicable disease transmission risk at the point of arrival to the health-care facility.^9^

High-income countries have tended to adopt five-tier triage tools, such as the Emergency Severity Index.^1^ These systems are not necessarily suited to low- and middle-income countries because of differing epidemiology, service demands and resource arrangements. Consistent with this, clinicians in low-resource settings have emphasized the need for triage instruments that are simple, efficient and reliable.^10^^,^^11^

Several context-specific triage scales have been developed for use in low-resource settings. An example is WHO’s Emergency Triage Assessment and Triage system, a three-tier, paediatric-focused tool that links with Integrated Management of Childhood Illness.^12^^–^^14^ The tool, which has been extensively studied across the African region,^12^^,^^13^ uses clinical discriminators, such as signs and symptoms, rather than physiologic parameters to define urgency. 

Another widely studied tool is the South African Triage Scale.^15^ This instrument has been implemented across various countries and contexts,^13^^,^^16^ including by *Médecins Sans Frontières*.^17^^,^^18^ The tool categorizes patients into four urgency levels based on presenting signs and symptoms as well as physiologic criteria, calculated as a triage early warning score.^15^

Several systematic reviews have considered the validity and reliability of triage scales in low- and middle-income countries^13^^,^^16^ and in high-resource settings,^19^^–^^21^ identifying common limitations, such as suboptimal sensitivity in detecting critical time-sensitive conditions, and a lack of compelling data supporting any one instrument.^13^^,^^16^^,^^19^ The overall quality of the evidence for paediatric triage has been assessed as poor, and heterogeneity in research methods limits the direct comparison of different systems.^13^

A major challenge in evaluating triage performance is the lack of a definitive measure of urgency.^2^ For this reason, studies commonly assess predictive validity using emergency department outcomes, such as admission, as surrogate measures. This approach is problematic, however, because the requirement for inpatient care does not necessarily correlate with time-sensitivity. 

Some researchers have attempted to work around these issues by reporting under- and over-triage rates, usually defined as the proportion of non-urgent patients who are admitted and urgent patients who are not, and comparing the results against pre-specified standards.^17^^,^^22^^,^^23^ However, many studies that have adopted this approach have benchmarked against American College of Surgeons Committee on Trauma guidelines, despite the fact that (i) recommendations for trauma triage do not necessarily apply to other disease categories; and (ii) the performance targets provided by an American society are unlikely to be appropriate in the context of low- and middle-income countries.

The coronavirus disease 2019 (COVID-19) pandemic highlighted the importance of triage in both high- and low-resource settings.^24^^–^^27^ This insight reflects the essential contribution of emergency care to the assessment and management of patients with severe acute respiratory infection, as well as broader surveillance and disease control efforts.^24^^,^^25^ Qualitative data from the WHO Western Pacific Region has specifically identified the value of efficient triage and patient flow processes as part of a comprehensive pandemic response strategy.^28^

The experience of the pandemic also reinforced the value of systematized acute care capacity in low- and middle-income countries, strengthening the case for universal access to timely and quality emergency care as well as the global implementation of evidence-based triage tools.^2^^,^^3^^,^^5^ Consistent with this, the WHO *Living guidance for clinical management of COVID-19* recommends that all health-care facilities utilize a standardized triage tool to assess patients, and lists the *Interagency Integrated Triage Tool* as an acceptable system.^29^ Here, we summarize recent evidence regarding triage in low- and middle-income countries, with a focus on this particular tool.^8^

## Interagency Integrated Triage Tool

### Origin and implementation

**Fig. 1:**
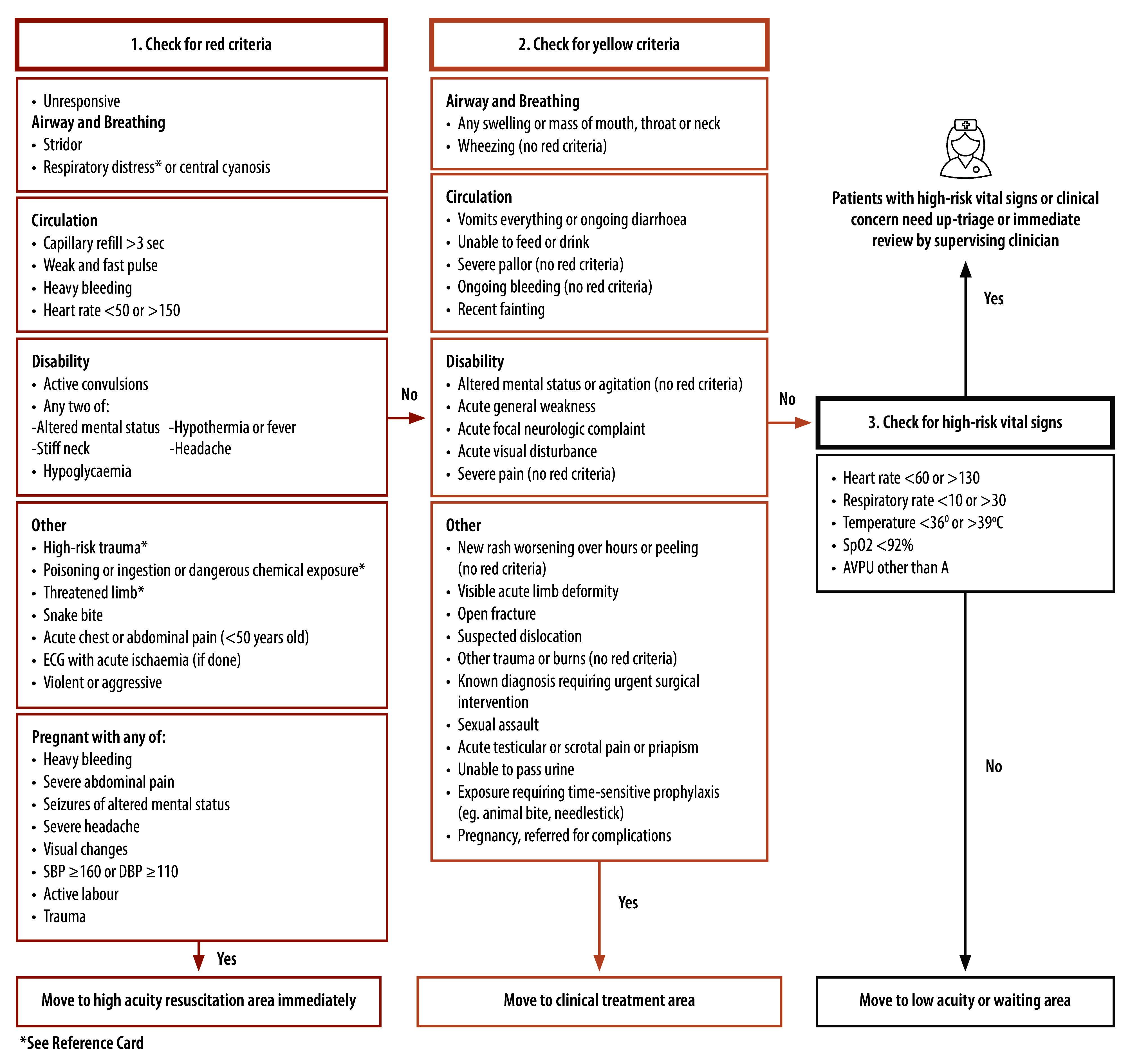
Interagency Integrated Triage Tool assessment criteria for patients aged 12 years and older

AVPU: alert, responding to voice, responding to pain or unresponsive; DBP: diastolic blood pressure; ECG: electrocardiogram; SBP: systolic blood pressure; SpO_2_: oxygen saturation; WHO: World Health Organization.

Note: asterisks are referring users to the reference card published in WHO *Clinical care of severe acute respiratory infections – tool kit*.^9^

Source: Adapted from WHO.^8^

The *Interagency Integrated Triage Tool *is a three-tier, colour-coded triage instrument developed collaboratively by WHO, *Médecins Sans Frontières* and the International Committee of the Red Cross (Fig. 1).^8^ Following its global release during the early stages of the COVID-19 pandemic, the tool has been promoted as part of WHO’s package of resources for strengthening emergency care systems.^30^

The triage tool leverages the strengths of *Emergency Triage Assessment and Treatment* and the *South African Triage Scale*, synergistically combining their most advantageous features (such as the traffic-light strategy of the former). While its clinical discriminators closely resemble those used in the *South African Triage Scale*, the tool eliminates the need to calculate a triage early warning score,^11^ instead relying on specific physiological parameters to identify time-critical patients (Fig. 1). This simplified approach is a key strength of the system.

The tool has recently been introduced in a variety of low- and middle-income countries, including Bangladesh, South Sudan and Vanuatu,^24^^,^^31^^–^^35^ and several implementation programmes have been developed to support health-care facilities in its adoption. For example, bespoke in-person training packages have been delivered in Honduras and Papua New Guinea as part of broader emergency care improvement programmes,^33^^,^^35^ and WHO and *Médecins Sans Frontières* have developed online learning tools for the system through the OpenWHO^36^ and Tembo^37^ platforms, respectively.

The only published assessments of *Interagency Integrated Triage Tool* implementation strategies come from Papua New Guinea.^33^^,^^38^ Reports from other countries are anticipated, but not yet available. Before the COVID-19 pandemic, a team of Australian and Papua New Guinean clinicians developed and delivered a 5-hour in-person education programme, coupled with peer mentoring by external nurses. Local health workers found this approach highly acceptable.^33^

The COVID-19 pandemic led to increased use of digital learning to assist with health-care system changes. In Papua New Guinea, this stimulated the development of a context-specific, smartphone-based online learning platform, including modules on the *Interagency Integrated Triage Tool* for emergency department staff.^39^^,^^40^ An evaluation of this approach identified improvements in knowledge and confidence among participants.^38^ Based on this evidence, digital learning combined with peer mentoring appears to be a feasible and effective strategy for implementing the tool.^38^ Lessons learnt through the change management process have been summarized elsewhere.^33^^,^^34^

### Performance characteristics

The predictive validity of the *Interagency Integrated Triage Tool* has been assessed across four emergency departments in Papua New Guinea, representing a mixture of urban and regional hospitals.^41^^–^^44^ The primary outcome in all studies was sensitivity for the detection of time-critical illness. At three sites, this measure was defined by a list of pre-specified diagnoses requiring urgent intervention, for example, ruptured ectopic pregnancy and acute myocardial infarction.^41^^–^^43^ Sensitivity was found to be 70.0% (95% confidence interval, CI: 50.6–85.3), 70.8% (95% CI: 58.2–81.4) and 77.8% (95% CI: 64.4–88.0) across the three emergency departments.^41^^–^^43^

The fourth study focused exclusively on patients with COVID-19, such that urgency was defined by the presence of severe or critical illness based on WHO criteria.^44^ In this evaluation, sensitivity (defined as the proportion of severe and critical COVID-19 patients who were allocated a red or yellow triage category on arrival at the emergency department) was found to be 74.6% (95% CI: 62.1–84.7), with a negative predictive value of 92.7% (95% CI: 88.4–95.8).^44^ Table 1 summarizes a broader set of test characteristics, related to the ability of the tool to predict admission and death at three of these study sites.

**Table 1:** Performance characteristics of the Interagency Integrated Triage Tool for predicting emergency department outcomes of admission and death, Papua New Guinea, 2019–2021^41^^–^^43^

Performance measure	Value of measure (95% CI)							
	ANGAU Memorial Provincial Hospital			Gerehu General Hospital			Mount Hagen Provincial Hospital	
	Admission	Death		Admission or transfer	Death		Admission	Death
Sensitivity, %	81.0 (72.7–87.7)	87.0 (66.4–97.2)		72.6 (67.5–77.2)	88.5 (69.8–97.6)		86.5 (81.4–90.7)	92.2 (81.1–97.8)
Specificity, %	66.3 (62.5–69.9)	60.5 (56.8–64.0)		81.2 (80.0–82.3)	77.5 (76.2–78.7)		84.6 (83.8–85.3)	83.2 (82.5–84.0)
Positive predictive value, %	30.2 (25.2–35.7)	6.43 (4.0–9.8)		24.1 (21.5–26.8)	2.2 (1.4–3.3)		12.2 (10.7–14)	3.0 (2.2–4.0)
Negative predictive value, %	95.1 (92.7–96.9)	99.3 (98.1–99.9)		97.3 (96.7–97.8)	99.9 (99.7–100.0)		99.6 (99.4–99.7)	99.9 (99.9–100.0)
Positive likelihood ratio	2.4 (2.1–2.8)	2.2 (1.8–2.6)		3.9 (3.5–4.2)	3.9 (3.4–4.6)		5.6 (5.2–6.0)	5.5 (5.0–6.0)
Negative likelihood ratio	0.3 (0.2–0.4)	0.2 (0.1–0.6)		0.3 (0.3–0.4)	0.2 (0.1–0.4)		0.2 (0.1–0.2)	0.1 (0.0–0.2)

CI: confidence interval.

Studies have also evaluated the inter-rater reliability of the tool, comparing agreement between a recently trained triage officer and an experienced, independent, external clinician. These studies have consistently demonstrated excellent agreement, with Cohen’s *κ* scores exceeding 0.8.^41^^–^^43^ Each study included a broad range of local clinicians, including community health workers with limited formal training. This is an important finding for a triage scale designed for low-resource settings, where specialist health workers are often scarce. The tool is also efficient, with reported median triage assessment times of 94–214 seconds.^41^^–^^43^ Speed of application is a relevant consideration for facilities facing high demand for emergency care.

### Acceptability

At each of the four health-care facilities in Papua New Guinea that have published their experience, the overwhelming majority of clinicians have expressed support for the tool and reported a positive impact on emergency department functioning (Table 2).^33^^,^^38^ The slightly lower ratings for Gerehu General Hospital may be explained by the challenging infrastructure and model of care in that department, which primarily operates as an outpatient facility with limited diagnostic and admission capacity.

**Table 2:** Acceptability of the Interagency Integrated Triage Tool as reported by surveyed emergency department clinicians, Papua New Guinea, 2019–2021^33^^,^^38^

Statement	No. Clinicians in agreement with statements (%)			
	ANGAU Memorial Provincial Hospital (*n* = 8)	Gerehu General Hospital (*n* = 24)	Mount Hagen Provincial Hospital (*n* = 15)	Port Moresby General Hospital (*n* = 22)
The new triage and flow system helps identify and prioritize the most urgent patients	8 (100.0)	24 (100.0)	15 (100.0)	22 (100.0)
The triage and flow system has improved patient flow in the emergency department	8 (100.0)	19 (79.2)	15 (100.0)	22 (100.0)
The triage assessment process is easy to follow	8 (100.0)	23 (95.8)	15 (100.0)	22 (100.0)
Implementation of the triage and flow system has improved my job satisfaction	8 (100.0)	21 (87.5)	15 (100.0)	21 (95.5)
Implementation of the triage and flow system has improved patient and staff safety in the emergency department	8 (100.0)	21 (87.5)	14 (93.3)	21 (95.5)

The results in Table 2 indicate that triage implementation can bring structure to an emergency department, and provide a foundation from which broader training and systems improvement initiatives can be developed. Based on this evidence, advantages of the system appear to be its ease of implementation and widespread acceptability.

### Interpreting evaluation data

Despite high levels of support among clinicians, the sensitivity of the tool to detect time-critical illness is not ideal.^41^^–^^44^ This drawback appears, however, to be a universal limitation of triage systems. A systematic review, reporting data predominantly from high-resource settings, identified sensitivities of 36–92% for a range of urgent conditions, including severe sepsis (36–74%) and ST-elevation myocardial infarction (56–92%).^19^ The absence of comparable data from low- and middle-income countries, for the *Interagency Integrated Triage Tool* or any other triage system, makes it difficult to benchmark the findings from Papua New Guinea.

The reported sensitivities also reflect that triage assesses urgency at a single point in time, for example on arrival at a health-care facility. Patients with acute illness or injury often demonstrate dynamic physiological changes, meaning those who present early might reasonably be expected to deteriorate. Consequently, criteria for a specific condition (for example severe pneumonia) may only be met later during a patient’s emergency department stay, such as at discharge when the diagnosis is recorded.

For this reason, no triage tool is likely to achieve 100% sensitivity without significantly compromising specificity. Rather, emergency care clinicians need to recognize the importance of repeat assessment (re-triage) for patients who are initially allocated a non-urgent category, and respond to clinical deterioration as appropriate. This approach to triage is essential, especially in an era of escalating demands on emergency departments. 

### Knowledge gaps 

While the Papua New Guinea experience provides important insights into the *Interagency Integrated Triage Tool*’s performance and acceptability, questions remain about the generalizability of these findings. The validity and reliability of triage tools, as measured through research and quality improvement initiatives, are widely understood to reflect the environments in which they are studied. Contextual factors, such as patterns of disease and resource availability, are likely to influence evaluation results.^20^ It is therefore critical to assess the tool in other countries and contexts. 

Future studies should explore the impact of the tool on a broader range of outcome measures, such as resource utilization and staff well-being. This recommendation reflects that triage has the potential to bring greater order to an emergency department, influencing functions beyond the assessment of urgency.

There is also a lack of data on community acceptance of the tool, particularly in settings where priority for health care is determined by factors other than urgency (such as social status). Additionally, further assessments are needed to evaluate the tool’s performance among specific patient groups, such as women and children, and in vulnerable populations, such as those affected by complex emergencies.

### Implications of WHO support

Despite the limitations of the available evidence, endorsement of the *Interagency Integrated Triage Tool* by WHO and other leading global health organizations makes its widespread implementation probable. Experience to date suggests that integrating the tool into WHO’s COVID-19 and emergency care toolkits has facilitated its adoption, partly due to the credibility associated with the WHO brand.^26^^,^^34^ Additionally, aligning the tool with other joint WHO and International Committee of the Red Cross resources, such as the Basic Emergency Care course, has helped frame triage as an essential component of a broader emergency care improvement strategy.^8^^,^^30^

In Papua New Guinea for instance, the adoption of the *Interagency Integrated Triage Tool* as the de facto national triage instrument is partly attributed to the trustworthiness of WHO and the incorporation of the tool into WHO guidance. Based on the data and experience presented above, a national training of trainers programme has been established, and local clinicians are increasingly implementing the tool across provincial hospitals.

From a global perspective, the positive early reception of the instrument presents both an opportunity and a challenge for its ongoing evaluation. Since the tool has already been disseminated by WHO, there may be barriers to further independent assessment of its performance. It is therefore critical that the global emergency care community commits to ongoing evaluation of the tool, alongside other components of the WHO emergency care systems toolkit.

## Future directions

### Measuring triage performance

With the growing number of triage tools available to emergency care providers in low- and middle-income countries, it is increasingly important to compare the performance of individual instruments and identify opportunities to enhance their sensitivity and specificity. However, the lack of standardized research methods for assessing triage systems remains problematic, limiting comparisons between tools. Experts have repeatedly identified the potential value of a universal approach to triage tool assessment, proposing a range of strategies and measures.^1^^,^^45^

Developing an agreed set of metrics requires consideration of the fundamental purpose of triage. Since the primary objective is to identify patients who are likely to benefit most from timely assessment and management, measuring sensitivity to detect sentinel time-critical diagnoses and/or the need for life-saving interventions, as has occurred in Papua New Guinea, has intrinsic value. This approach aligns with a previous proposal to use triage footprints for specific conditions to compare the performance of a given tool between settings.^1^ The strategy could also be adapted to compare the validity of different systems.

### Data-driven quality improvement

Many health-care facilities in low- and middle-income countries face challenges in achieving continuous quality improvement for triage and other emergency care functions. Appropriately, triage performance has been recommended as a core indicator of safety and quality in low-resource settings.^46^ Addressing this measure requires individual facilities to establish clear targets and systematically collect data on the timeliness and outcomes of care. The barriers to implementing these practices in low-resource settings are well documented.^46^^–^^48^

Registries represent a valuable tool for capturing and aggregating emergency department performance data, but have limited uptake in low- and middle-income countries.^48^ Experience from the WHO Western Pacific Region, including through the *Interagency Integrated Triage Tool* studies explored above, has demonstrated the feasibility of introducing simple, low-cost electronic registry systems to enable routine triage data collection.^34^ The WHO Clinical Registry represents a potential vehicle for addressing this issue, subject to the provision of data entry capabilities and resources.^30^

For the *Interagency Integrated Triage Tool*, developing an implementation, monitoring and evaluation toolkit would help to ensure consistent application of the system and facilitate ongoing quality improvement. Ideally, this toolkit should be developed at a global level and made available through an open access arrangement for facilities, organizations and authorities seeking to introduce the tool.

### Triage for improving outcomes

Although there is substantial evidence supporting the acceptability, validity and reliability of triage scales in low- and middle-income countries, the literature is limited regarding the impact of triage implementation on clinical outcomes and process measures.^2^ This paucity of data reflects the challenges of conducting high-quality research in low-resource emergency care settings.^47^

Fundamentally, triage is aimed at expediting the care of patients with urgent care needs. Although the triage process serves additional functions, such as bringing structure to an emergency department and defining resource requirements, there is a strong imperative to establish an empirical evidence base for its impact on clinical outcomes.

Intuitively, implementing a system designed to identify and prioritize time-critical patients should translate to better care. Supporting this is a substantial body of evidence linking timely management to improved health-care outcomes for various conditions, such as myocardial infarction, trauma and sepsis.^2^ A key question, however, is whether the introduction of structured approaches to triage, such as the Interagency Integrated Triage Tool, conveys an advantage over informal or intuition-based systems.^49^

A systematic review conducted in 2023 attempted to answer this question, identifying 16 studies that used pre-post methods to assess the impact of triage implementation in low- and middle-income countries.^50^ Effect measures in these studies included mortality, length of stay, waiting time, patient satisfaction and admission rates. Of these, early mortality and time to clinician assessment were evaluated most frequently, and most studies using these outcomes identified a reduction in the number of deaths and waiting time. The quality of the evidence was moderate for these measures, but low or very low for all other process indicators and outcomes.^50^

Although the available data suggest that triage implementation is likely to improve quality of care, there is a need for further research and more robust study methods to control the significant risks of publication bias and confounding. This work should be considered a priority for the global emergency care community, particularly in relation to the *Interagency Integrated Triage Tool*. Studies using stepped-wedge design are well suited to this purpose.

## Conclusion

Emergency care facilities in low- and middle-income countries implementing a structured triage system have a range of tools to choose from. Local stakeholders should select an instrument that is applicable to the patient population, supported by evidence and acceptable to the community. 

The *Interagency Integrated Triage Tool* is likely to meet these criteria in many countries and contexts. Although it is not a perfect triage instrument,^49^ the available data suggest it is valued by clinicians, feasible to implement with limited digital or in-person training and can be efficiently applied by triage officers.^33^^,^^38^^,^^41^^–^^44^ Importantly, the tool’s predictive validity (regarding admission and mortality) and inter-rater reliability (reflecting clinician agreement) appear comparable to, if not superior to, other triage tools designed for low- and middle-income countries.

Despite the intuitive and widely acknowledged value of triage, establishing high-quality evidence of its impact on quality of care is a critical next step. Further research will support WHO efforts to strengthen emergency care systems globally.
